# RISK FACTORS FOR POSTOPERATIVE PANCREATIC FISTULA FOLLOWING PANCREATICODUODENECTOMY: TUNISIAN CENTER EXPERIENCE

**DOI:** 10.1590/0102-6720202500008e1877

**Published:** 2025-04-14

**Authors:** Nizar Khedhiri, Haithem Zaafouri, Wael Boujelbene, Mouna Cherif, Imen Helal, Meryam Mesbahi, Dhafer Haddad, Anis Ben-Maamer

**Affiliations:** 1 Habib Thameur Hospital, General Surgery – Tunis, Tunísia; 2 Habib Thameur Hospital, Pathology Department – Tunis, Tunísia; 3 Tunis El Manar University, Faculty of Medicine of Tunis – Tunis, Tunísia.

**Keywords:** Pancreatic Fistula, Pancreaticoduodenectomy, Morbidity, Fístula Pancreática, Pancreaticoduodenectomia, Morbidade

## Abstract

**BACKGROUND:**

Pancreaticoduodenectomy (PD) is a major intervention in digestive surgery. Although its mortality is currently low in experienced centers, morbidity remains high, dominated by a pancreatic fistula.

**AIMS:**

The aim of this study was to analyze the risk factors for postoperative pancreatic fistula (POPF) after PD.

**METHODS:**

A retrospective study was conducted at the General Surgery Department of Habib Thameur University Hospital in Tunis for 12 years (2010–2021). All patients who underwent PD were included regardless of indications.

**RESULTS:**

Our series comprised 50 patients, consisting of 27 men and 23 women. The rate of a pancreatic fistula was 32% (16 patients) with an average time of onset of 5 days (1–12 days). It was observed as a biochemical leak (grade A) in 1 patient (2%), pancreatic fistula grade B in 5 patients (10%), and pancreatic fistula grade C in 10 patients (20%). Pancreatic fistula was responsible for 10% of postoperative mortality (five patients). Univariate analysis showed a statistically significant correlation between POPF and the following factors: diameter of the main pancreatic duct ≤3 mm (p=0.036, p<0.05), soft texture of the pancreas (p=0.025, p<0.05), pancreaticojejunostomy by two semi-overlapping sutures (p=0.049, p<0.05), and fasting blood glucose level ≤8 mmol/l (p=0.025, p<0.05). Multivariate analysis showed that soft pancreatic texture was the only independent risk factor for POPF (p=0.02, p<0.05).

**CONCLUSION:**

The soft texture of the pancreas is the only independent risk factor for POPF. Prospective randomized studies are still needed to accurately determine the true risk factors for a pancreatic fistula after PD.

## RESUMO

**RACIONAL:**

A pancreaticoduodenectomia (PD) é uma intervenção importante na cirurgia digestiva. Embora sua mortalidade seja atualmente baixa em centros experientes, a morbidade permanece alta, dominada pela fístula pancreática.

**OBJETIVOS:**

Analisar os fatores de risco para fístula pancreática pós-operatória (FPO) após pancreaticoduodenectomia.

**MÉTODOS:**

Foi realizado um estudo retrospectivo no Departamento de Cirurgia Geral do Hospital Universitário Habib Thameur, em Túnis, durante 12 anos (2010–2021). Todos os pacientes submetidos à pancreaticoduodenectomia foram incluídos, independentemente da indicação.

**RESULTADOS:**

Nossa série incluiu 50 pacientes, sendo 27 homens e 23 mulheres. A taxa de fístula pancreática foi de 32% (16 pacientes) com um tempo médio de início de 5 dias (1-12 dias). Foi um vazamento bioquímico (grau A) em 1 paciente (2%), fístula pancreática grau B em 5 pacientes (10%) e grau C em 10 pacientes (20%). A fístula pancreática foi responsável por 10% da mortalidade pós-operatória (5 pacientes). A análise univariada demonstrou uma correlação significativa entre a fístula pancreática pós-operatória e os seguintes fatores: diâmetro do ducto pancreático principal ≤3 mm (p=0,036, p<0,05), textura macia do pâncreas (p=0,025, p<0,05), pancreaticojejunostomia por 2 suturas sobrepostas pela metade (p=0,049, p<0,05), glicemia de jejum ≤8 mmol/l (p=0,025, p<0,05). A análise multivariada mostrou que a textura macia do pâncreas foi o único fator de risco independente para fístula pancreática pós-operatória (p=0,02, p<0,05).

**CONCLUSÕES:**

A textura macia do pâncreas é o único fator de risco independente para FPO. Ainda são necessários estudos prospectivos e randomizados para determinar com precisão os verdadeiros fatores de risco para fístula pancreática após a PD.

## INTRODUCTION

Pancreaticoduodenectomy (PD) is a major intervention in digestive surgery that corresponds to the monobloc removal of the head of the pancreas, the common bile duct, the duodenum, and often the distal part of the stomach. Tumors of the biliopancreatic junction represent this procedure's main indication and constitute the only gesture with a curative aim. Although its mortality has significantly decreased in recent years, its morbidity remains high despite advances in surgical technique, anesthesia, and interventional radiology^15^.

Postoperative pancreatic fistula (POPF) remains the most difficult challenge after PD, even in specialized units, and its occurrence remains the main contributor to postoperative morbidity and mortality. It is the most serious complication, with an incidence that varies from 11.4 to 64.3% according to different studies. Recent studies have revealed that many factors, including pre-operative, intraoperative, and postoperative, influence the development of POPF^10,14,16,17,23,31^.

The objectives of our study were to determine the incidence of a pancreatic fistula after PD, analyze the risk factors for POPF, and compare our results with recent literature.

## METHODS

### Patients and data collection

This retrospective analytical study was conducted over 12 years from January 1, 2010, to December 31, 2021. The clinical cases were collected from the General Surgery Department of Habib Thameur Hospital in Tunis. This study included all patients who had undergone PD for any indication and was approved by the Ethics Committee of the Institution (HTHEC-2024-16). A complete analytical file was done for each PD case, containing essentially the following information: age, sex, American Society of Anesthesiologists score, medical history, hemoglobin level, liver function tests, blood sugar levels, nutritional status, biological markers of inflammation, intraoperative findings (diameter of the main bile duct, diameter of the duct of Wirsung, state of the pancreatic parenchyma, type of pancreaticodigestive anastomosis, bilio-digestive anastomosis, gastro-jejunal anastomosis, associated gestures, intraoperative incidents, duration of intervention, and intraoperative transfusions), immediate follow-up (mortality, overall morbidity, non-specific morbidity, specific morbidities such as a pancreatic fistula, as well as date of occurrence and treatment or repeat surgery). The judgment criteria consisted of studying the following elements:

1. Operative mortality: The occurrence of death within 30 days of the operation or during the same hospitalization, irrespective of its duration.
2. POPF (according to the International Study Group for Pancreatic Surgery [ISGPS] in 2016): Based on the literature since 2005, a clinically relevant POPF is now redefined as the drainage of any measurable volume of fluid with amylase activity >3 times the upper limit of the institutional normal serum amylase activity, associated with a direct alteration of the clinical condition related to the POPF. Therefore, the former "grade A POPF" is now redefined and referred to as a "biochemical leak" because it has no clinical significance and is no longer designated as a true POPF.

Grade B and C POPFs are confirmed but more narrowly defined: grade B requires the modification of postoperative management; drains are left in place for >3 weeks or repositioned via endoscopic or percutaneous procedure. Grade C refers to POPFs that require re-intervention or result in organ failure and/or mortality attributable to the POPF^4,12,30^.

### Statistical analysis

The results were entered and analyzed using SPSS software version 22.0. Qualitative variables were expressed as percentages and quantitative variables as mean±standard deviation or median value and extremes. Qualitative values were compared using a chi-square test. Quantitative values were compared using the Student's t-test or Mann-Whitney test. Risk factors for a pancreatic fistula were assessed by univariate statistical analysis and p<0.05 was considered statistically significant. Variables with p<0.15 were included in multivariate logistic regression analysis to test for independent risk factors for POPF.

## RESULTS

This epidemiological study was conducted on 50 cases of PD that were collected from the General Surgery Department of Habib Thameur Hospital, Tunis, between January 1, 2010, and December 31, 2021. Our series included 27 men (54%) and 23 women (46%), with a sex ratio of 1.17. The mean age in our series was 57.18±10.97 years, with extremes ranging from 14 to 75 years. The indication for PD was tumors of the head of the pancreas in 46% of cases, tumors of the ampulla of Vater in 28% of cases, cholangiocarcinoma of the lower bile duct in 18% of cases, and tumors of the duodenum in 8% of cases. The mortality rate was 16% (8 patients). The overall morbidity rate was 82%. Non-specific complications occurred in 10 patients (20%) (Table 1) and specific complications in 40 patients (80%) (Table 2).

**Table 1:** Complications after pancreaticoduodenectomy.

Complications	Number	Frequency (%)
Pulmonary embolism	1	2
Pulmonary infection	3	6
Urinary tract infection	3	6
Coronary syndrome	1	2
Diabetes imbalance	3	6

**Table 2:** Surgical complications after pancreaticoduodenectomy.

Complications	Number	Frequency (%)	Management
Wall infection	6	12	Medical treatment
Post-operative hemorrhage	10	20	Medical treatment: 8 patients Surgical treatment: 2 patients
Pancreatic fistula	16	32	Medical treatment: 6 patients Surgical treatment: 10 patients
Biliary fistula	3	6	Medical treatment: 2 patients Surgical treatment: 1 patient
Digestive fistula	4	8	Medical treatment: 3 patients Surgical treatment: 1 patient
Gastroparesis	6	12	Medical treatment

### Postoperative pancreatic fistula

The rate of POPF was 32% (16 patients) with an average time of onset of 5 days, ranging from 1 day to 12 days. The incidence of fistula was as follows: a biochemical leak (formerly called grade A) in 1 patient (2%), grade B pancreatic fistula in 5 patients (10%), and grade C pancreatic fistula in 10 patients (20%).

Pancreatic fistula was responsible for 10% of postoperative mortality (5 patients). The median time to postoperative death in cases of a severe pancreatic fistula was 21 days, with extremes ranging from 6 days to 36 days.

The diagnosis of a pancreatic fistula was suspected in all cases by the amber appearance of the drainage fluid, confirmed in 6 cases by the determination of amylase in the drainage fluid (greater than three times the sérum amylase activity) and in 10 cases by intraoperative exploration in the event of surgical revision.

In the case of grade B pancreatic fistula, medical treatment involved extending the use of sandostatin, maintaining or repositioning the drainage, and administering antibiotics to the patient. The average time for resolution was 10 days, ranging from 5 days to 18 days.

In the case of grade C pancreatic fistula, we opted for surgical revision in all cases. In four cases, the indication was the development of acute generalized peritonitis, and in six cases, the failure of medical treatment with the development of multi-visceral failure.

Several variables were studied to identify risk factors for the occurrence of POPF.

### Univariate analysis of risk factors for pancreatic fistulas

Univariate analysis did not show a significant correlation between POPF and the following factors: gender, age, history of diabetes, abdominal surgery, smoking, body mass index, total bilirubin, albumin, preoperative biliary drainage, histological type of tumor, intraoperative transfusion, and type of pancreaticodigestive anastomosis (pancreáticojejunostomy in all our patients): end-to-side or end-to-end anastomosis (Table 3). However, a significant correlation was observed with the following factors: diameter of the pancreatic duct ≤3 mm, soft texture of the pancreas, non-administration of sandostatin, pancreaticojejunostomy by two semi-overlapping sutures, and fasting blood glucose level ≤8 mmol/l.

**Table 3:** Risk factors for pancreatic fistulas according to univariate analysis.

Variable		Pancreatic fistula (%)	No pancreatic fistula (%)	p-value
Gender				
	Male	7 (43.8)	20 (58.8)	0.373
	Female	9 (56.2)	14 (41.2)	
Age (years)				
	<65	12 (75)	25 (73.5)	1
	≥65	4 (25)	9 (26.5)	
Diabetes				
	No	11 (68.75)	23 (67.6)	1
	Yes	5 (31.25)	11 (32.4)	
Fasting blood glucose level (mmol/l)				
	≤8	11 (84.6)	16 (47.1)	0.025
	>8	2 (15.4)	18 (52.9)	
Previous abdominal surgery				
	No	12 (75)	31 (91.2)	0.190
	Yes	4 (25)	3 (8.8)	
Smoking history				
	No	11 (68.8)	18 (52.9)	0.365
	Yes	5 (31.2)	16 (47.1)	
BMI				
	<25	9 (75)	18 (66.7)	0.719
	≥25	3 (25)	9 (33.3)	
Total bilirubin (μmol/l)				
	<250	8 (53.3)	22 (66.7)	0.522
	≥250	7 (46.7)	11 (33.3)	
Albumin				
	<35	8 (63.6)	14 (72.7)	0.709
	≥35	3 (36.4)	8 (27.3)	
Wirsung diameter (mm)				
	≤3	11 (68.75)	12 (35.3)	0.036
	>3	5 (31.25)	22 (64.7)	
Preoperative biliary drainage				
	No	11 (68.75)	26 (76.5)	0.731
	Yes	5 (31.25)	8 (23.5)	
Pancreatic consistency				
	Soft	11 (68.75)	5 (14.7)	<0.001
	Hard	5 (31.25)	29 (85.3)	
Histological type (pathology specimen)				
	Pancreatic head tumor	6 (37.5)	15 (44.1)	0.878
	Ampulloma	5 (31.3)	9 (26.5)	
	Lower bile duct tumor	4 (25)	5 (14.7)	
	Duodenal tumor	1 (6.3)	3 (8.8)	
	Pancreatic pseudocyst	0 (0)	2 (5.9)	
Intraoperative transfusion				
	No	14 (87.5)	26 (76.5)	0.468
	Yes	2 (12.5)	8 (23.5)	
Intraoperative sandostatin				
	No	5 (31.25)	1 (2.9)	0.01
	Yes	11 (68.75)	33 (97.1)	
Surgical technique: anastomosis				
	End to side	12 (75)	30 (88.2)	0.249
	End to end	4 (25)	4 (11.8)	
	Overlapping sutures	8 (50)	7 (20.6)	0.049
	Interrupted stitching	8 (50)	27 (79.4)	

BMI: body mass index.

### Multivariate analysis

The study identified several risk factors for pancreatic fistulas through univariate analysis, including pancreatic duct diameter, pancreatic texture, administration of sandostatin, pancreaticojejunostomy with overlapping sutures, and fasting blood glucose level (≤8 mmol/l). These factors were further analyzed using multivariate analysis (logistic regression) to determine their independent contributions to the risk of developing a pancreatic fistula postoperatively.

The results of the multivariate analysis indicated that among all the factors considered, only the texture of the pancreas was a significant independent risk factor. Specifically, a soft texture of the pancreas was associated with a high odds ratio (OR) of 42.65, with a p value of 0.02, indicating statistical significance (p<0.05). This suggests that patients with a softer pancreatic texture are significantly more likely to develop a pancreatic fistula following surgery compared to those with a firmer texture, highlighting the importance of pancreatic texture in surgical outcomes.

For more detailed insights, refer to Table 4, which presents the comprehensive results of the analysis.

**Table 4:** Risk factors for pancreatic fistulas according to multivariate analysis.

Variable	p-value	Odds ratio (confidence interval)
Wirsung diameter ≤3 mm	0.239	0.044 (0.001–1.525)
Soft pancreatic consistency	0.02	42.65 (1.819–1000.236)
Preoperative sandostatin	0.098	641.81 (0.301–1368754.677)
Pancreaticojejunal anastomosis with overlapping sutures	0.233	0.110 (0.003–4.136)
Fasting blood glucose level ≤8 mmol/l	0.084	0.044 (0.001–1.525)

## DISCUSSION

Pancreatic surgery is one of the visceral surgeries with the highest mortality and morbidity rates. This is due to the anatomical and histological characteristics of the pancreas, the aggressive nature of the pathology affecting the organ, and the technical difficulties associated with surgery^20^.

POPF remains the most difficult challenge after PD, even in specialized units, and its occurrence remains the main contributor to postoperative morbidity and mortality^19,31^.

Recent studies have revealed that many preoperative, intraoperative, and postoperative factors influence the occurrence of a POPF.

### Soft pancreatic consistency

In several studies, the soft texture of the pancreas has been widely recognized as an important risk factor for pancreatic fistulas^13,24^. Ke et al. retrospectively analyzed 170 cases of PD and concluded that the risk of developing a pancreatic fistula in patients with a soft pancreas was 5,257 times higher than that in patients with a hard pancreas^17^. Hu et al. retrospectively analyzed 539 cases of PD to identify the risk factors for POPF^14^. In their study, 402 patients had a soft pancreas (POPF rate: 56.72%) and 137 patients had a hard pancreas (POPF rate: 29.93%). Univariate analysis and multivariate logistic regression analysis showed that the difference in POPF rates was statistically significant (p=0.000, p<0.05), suggesting that patients with a soft pancreas had a higher risk of developing a pancreatic fistula after PD than patients with a hard pancreas with an OR of 3.048^13,14^. Similarly, in a multicentric analysis of 11 Japanese medical institutions (1239 patients), Kawai et al. showed that a soft pancreas (OR=2.7, p=0.001, p<0.05) is a significant predictor of clinical pancreatic fistula^16^.

A multicentric study by the French Association of Surgery confirmed that the rate of a pancreatic fistula was significantly higher in patients with normal or soft pancreatic parenchyma^1^. Our study is in agreement with previous studies: Indeed, 16 patients had a soft pancreas (pancreatic fistula rate, 68.75%) and 34 patients had a hard pancreas (pancreatic fistula rate, 14.7%). Univariate analysis showed a statistically significant difference between the two groups (p<0.001), suggesting that patients with soft pancreatic parenchyma were more likely to develop a POPF after PD than patients with a hard pancreas. Furthermore, multivariate logistic regression analysis indicated that a soft pancreas was an independent risk factor for the development of a pancreatic fistula. The OR (42.65; 95% confidence interval [CI]=1.819–1000.236) demonstrated that the risk of developing a pancreatic fistula in patients with a soft pancreas was 42.65 times higher than that in patients with a hard pancreas.

Ke et al. explained the causes of a pancreatic fistula after PD in patients with a soft pancreas. Indeed, the parenchymal tissue of the normal pancreas is fragile and contains abundant pancreatic ducts related to its exocrine function. When performing a pancreaticodigestive anastomosis with a soft pancreas, the fragile pancreatic tissue and thin pancreatic ducts can be easily cut during suturing and knotting, which can lead to a pancreatic fistula^17^.

### Thin main pancreatic duct

Pancreatic duct diameter of 3 mm is a risk factor for a pancreatic fistula after PD according to several studies^14,17,22,35^. Hu et al. showed that the difference in POPF rates was statistically significant (p=0.000, p<0.05 with OR=2.062), suggesting that patients with a pancreatic duct diameter of 3 mm had a higher risk of developing a pancreatic fistula after PD than patients with a pancreatic duct diameter >3 mm^14^. In our series of patients, univariate analysis also showed that the incidence of a pancreatic fistula after PD was statistically significantly higher in patients with a main pancreatic duct diameter of 3 mm (68.75% vs. 31.25%; p=0.036, p>0.05). However, multivariate analysis showed that this difference was not statistically significant (p=0.239, p>0.05).

The lower incidence of a pancreatic fistula after PD in patients with a pancreatic duct >3 mm may be related to prolonged obstruction of the pancreatic duct, leading to dysfunction of exocrine pancreatic function, pancreatic duct fibrosis, pancreatic fibrosis, ease of suturing, and lower risk of pancreatic duct injury during suturing and knot tying^14^.

### Surgical techniques

An important role in the development of a pancreatic fistula after pancreatic duodenectomy (PD) has been attributed to the choice of pancreaticodigestive anastomosis^16,24,25,29^. The benefit of pancreaticogastric anastomosis compared with pancreaticojejunal anastomosis remains a subject of debate. Some comparative studies, meta-analyses, and multicentric studies have demonstrated a significantly lower rate of pancreatic fistulas after pancreaticogastric anastomosis^1,8,26^. However, other studies have not found a significant difference between the two types of anastomosis^3,7^. Pancreaticogastric anastomosis offers a number of advantages that reduce the risk of fistula, such as the proximity of the stomach to the remaining pancreas, which reduces tension on the anastomosis; the rich vascularity of the stomach, which reduces the risk of anastomotic ischemia; and the acidic gastric environment, which inhibits the activation of pancreatic enzymes. However, the use of pancreaticogastric anastomosis has a major disadvantage, which is an increased risk of postoperative bleeding^18^. In a clinical study that observed the long-term effects of pancreatico-enteric anastomosis, Benini et al. found that exocrine pancreatic function was significantly more affected after pancreaticogastric anastomosis and was also associated with decreased vitamin D levels and fat malabsorption^5^.

For these reasons, the approach adopted by their surgeons was the Child technique, which is the oldest technique: pancreaticojejunal anastomosis was preferred in our surgical department^25^.

Pancreaticojejunal anastomosis is a critical step in PD and can affect the surgical outcome. Indeed, pancreaticojejunal anastomosis is a complex procedure, and several reconstruction techniques have been developed to reduce the risk of pancreatic fistulas. In our series, the type of pancreaticojejunal anastomosis, end-to-side or end-to-end, was not a statistically significant risk factor (p=0.249, p>0.05) for pancreatic fistulas. The role of intussusception and intubation techniques of the Wirsung duct in the development of pancreatic fistulas cannot be studied, given the limited number of cases using these two techniques (two cases of intussusception and three cases of intubation of the Wirsung duct). Several techniques for pancreaticojejunal anastomosis have been published in the literature in the last decade. Poon et al. found that duct-to-mucosal anastomosis was a safer technique than intussusception anastomosis^29^.

Marcus et al. found that duct-to-mucosal anastomosis was associated with a low rate of pancreatic fistulas in low-risk patients (with a dilated pancreatic duct or fibrous pancreas), whereas the end-to-end intussusception technique was safer in high-risk patients (with small ducts or a soft, friable pancreas)^24^.

Yang et al. found in their series that the pancreatic fistula rate was 6.25% in patients who had a pancreaticojejunal duct-to-mucosal anastomosis, compared with 19.6% in the intussusception group^35^. In the study by Hu et al., a double-layer pancreaticojejunal anastomosis (mucosa–mucosa and pancreas-jejunum) was performed in 398 patients (POPF rate: 57.54%) and a single-layer pancreaticojejunal mucosa-mucosa anastomosis was performed in 141 patients (POPF rate: 35.46%). Univariate and multivariate analyses showed that the difference was statistically significant (p=0.001, p<0.05), suggesting that double-layer pancreaticojejunal anastomosis was a risk factor for pancreatic fistulas after PD with an OR equal to 2.102^14^.

Several retrospective studies have reported a very low incidence of postoperative grade B/C pancreatic fistulas after pancreaticojejunal anastomosis with the Blumgart mattress suture technique and reported its superiority over the interrupted suture technique^11,27^. Interrupted suturing of the pancreatic parenchyma and jejunal seromuscular layer may generate tangential shear forces while tightening the knots, and the suture material can easily tear the pancreatic tissue. The Blumgart method, on the other hand, has the advantage of avoiding shear stresses on the pancreas using the mattress suture technique^11,27^.

The use of internal stents in pancreaticojejunal anastomosis is another controversial topic, but most publications have not considered it an advantageous technique^28^.

### Diabetes and fasting glucose level

A meta-analysis of 16 observational clinical studies revealed that diabetes was associated with a reduced risk of POPF (p=0.01, p<0.05). In contrast, patients without diabetes had a higher risk of developing a POPF because their pancreas had more adipose tissue and the pancreas was soft^34^. Mathur et al. also concluded that patients with diabetes may have less fat and more pancreatic fibrosis, which may protect them from the occurrence of a POPF after PD^25^.

### Role of somatostatin and its analogs

Somatostatin analogs are currently used to prevent the development of POPF. However, their use is controversial^2^.

In our series, univariate analysis showed that the incidence of a pancreatic fistula after PD was statistically significantly lower in patients receiving intraoperative and postoperative sandostatin (p=0.01, p<0.05). However, multivariate analysis showed that this difference was not statistically significant (p=0.098, p>0.05).

Randomized controlled trials of somatostatin and somatostatin analogs after pancreatic surgery have been performed, with conflicting results. Some have reported that prophylactic somatostatin octreotide significantly reduced the incidence of pancreatic fistulas after PD^10,32^. Others have reported that the use of somatostatin analogs, including octreotide and vapreotide, did not reduce pancreatic fistulas after pancreatic surgery^9,33^.

Furthermore, available meta-analyses provide conflicting results regarding the beneficial effects of somatostatin and its analogs for preventing POPF. A recent meta-analysis of 15 studies involving 2,221 patients showed that somatostatin prophylaxis reduced the incidence of POPF after all types of pancreatic resections. There was no evidence of a reduction in mortality^21^. Another recent meta-analysis, including 12 randomized trials with 1,615 patients after pancreaticoduodenectomy, concluded that the somatostatin analog did not statistically significantly reduce the incidence of POPF (OR=0.48; 95%CI 0.22–1.06, p=0.07, p>0.05)^2^.

The conflicting results of previous studies have led to many different protocols for the use of somatostatin and its analogs. Thus, Bootsma et al.^6^ performed a national analysis comparing different protocols using somatostatin and its analogs and their effects on POPF levels. This analysis suggests that the administration of lanreotide in all patients undergoing PD is associated with a reduced rate of POPF (p=0.015, p<0.05) compared with other protocols. Furthermore, in a sub-analysis of patients at high risk for POPF, the lanreotide protocol had a significantly lower rate of pancreatic fistulas. The results of using the octreotide protocol in patients at high risk for pancreatic fistulas (soft pancreas and thin Wirsung's duct) were comparable with those of the lanreotide protocol.

In addition, the use of somatostatin analogs had no significant impact on mortality.

## CONCLUSIONS

Our study provided valuable information on the risk factors associated with POPF after PD. According to univariate analysis, we found that several factors, including main pancreatic duct diameter ≤3 mm, soft texture of the pancreas, pancreatojejunostomy with two semi-overlapping sutures, and fasting blood glucose level ≤8 mmol/l, were associated with a high rate of POPF. Multivariate analysis showed that the soft texture of the pancreas was the only independent risk factor for POPF. Intraoperative administration of sandostatin appeared to protect against the occurrence of pancreatic fistulas.

These findings highlight the importance of the careful selection of patients and surgical technique to minimize the risk of POPF in PD procedures. Although further prospective randomized studies are needed to confirm these risk factors and refine preventive strategies, our study contributes to the growing body of knowledge aimed at improving the outcomes of this complex surgical procedure.

Central Message Pancreaticoduodenectomy (PD) is a major intervention in digestive surgery that corresponds to the monobloc removal of the head of the pancreas, the common bile duct, the duodenum, and often the distal part of the stomach. Postoperative pancreatic fistula (POPF) remains the most difficult challenge after PD, even in specialized units, and its occurrence remains the main contributor to postoperative morbidity and mortality. It is the most serious complication, with an incidence that varies from 11.4 to 64.3% according to different studies. Recent studies have revealed that many preoperative, intraoperative, and postoperative factors influence the development of POPF.

Perspectives Univariate analysis showed that several factors, including the diameter of the main pancreatic duct ≤3 mm, soft texture of the pancreas, pancreaticojejunostomy by two semi-overlapping sutures, and fasting blood glucose level ≤8 mmol/l, were associated with a high rate of postoperative pancreatic fistula (POPF). Multivariate analysis showed that soft pancreatic texture was the only independent risk factor for POPF. Intraoperative administration of sandostatin seemed to protect against the occurrence of a pancreatic fistula.
